# Phytochemical Characterization of Wild Hops (*Humulus lupulus* ssp. *lupuloides*) Germplasm Resources From the Maritimes Region of Canada

**DOI:** 10.3389/fpls.2019.01438

**Published:** 2019-12-11

**Authors:** Jason L. McCallum, Mark H. Nabuurs, Spencer T. Gallant, Chris W. Kirby, Aaron A. S. Mills

**Affiliations:** Agriculture and Agri-Food Canada, Charlottetown Research and Development Centre, Charlottetown, Canada

**Keywords:** *Humulus lupulus* L., *lupuloides*, wild hops, chemotaxonomy, kaempferol-3-O-(6’’-O-malonyl)-β-D-glucopyranoside, UPLC-MS, NMR

## Abstract

A survey was conducted in the Maritimes region of eastern Canada to measure the phytochemical diversity of prenylchalcone, soft resins (alpha & beta acids), and flavonol constituents from 30 unique wild-growing populations of hops (*Humulus lupulus* L.). Based on cone chemometrics, the majority of accessions (63.3%) are native *Humulus lupulus* ssp. *lupoloides*, with cones containing both xanthogalenol and 4’-*O*-methyl xanthohumol as chemotaxonomic indicator molecules. Interestingly, the leaves of all verified *Humulus lupulus* ssp. *lupulus* accessions accumulated high proportions (>0.20 total flavonols) of two acylated flavonol derivatives (kaempferol-3-*O*-(6’’-*O*-malonyl)-β-D-glucopyranoside; quercetin-3-*O*-(6’’-*O*-malonyl)-β-D-glucopyranoside), both previously unreported from hops leaves. The native *lupuloides* accessions examined possess only trace amounts of this compound in their leaves (<0.10 total flavonols), suggesting its potential utility as a novel, leaf-derived chemotaxonomic marker for subspecies identification purposes. A leaf-derived taxonomic marker is useful for identifying wild-growing accessions, as leaves are present throughout the entire growing season, whereas cones are only produced late in summer. Additionally, the collection of cones from 10-meter tall wild plants in overgrown riparian habitats is often difficult. The total levels of alpha acids, beta acids, and prenylchalcones in wild-collected Maritimes *lupuloides* cones are markedly higher than those previously reported for *lupuloides* individuals in the westernmost extent of its native range and show potentially valuable traits for future cultivar development, while some may be worthy of immediate commercial release. The accessions will be maintained as a core germplasm resource for future cultivar development.

## Highlights

- 30 wild-growing Maritimes Hops accessions were analyzed
- 63% were characterized as *Humlulus lupulus* ssp. *lupuloides*
- Maritimes *lupuloides* present different chemical profiles than previously characterized populations in western Great Plains region

## Introduction

**Figure 1:**
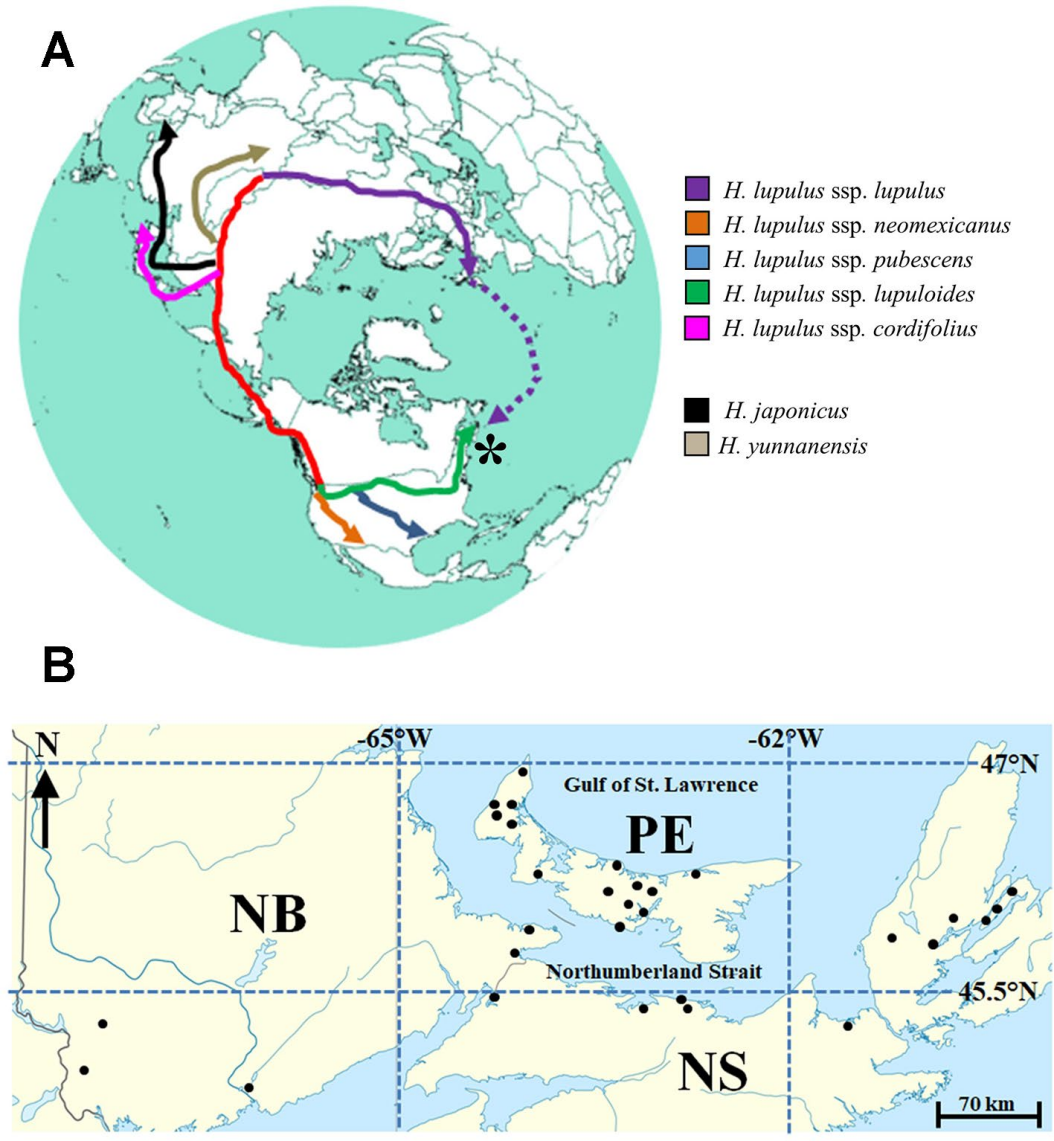
**(A)** Reconstructed circumpolar distribution of *Humulus lupulus* L., from purported central Asian origins. Migration and colonization of the Maritimes region of Canada (*) by *H. lupulus* ssp. *lupuloides* (green) possibly represents the farthest geographic divergence from the origin of species. Dotted purple line represents recent introduction *via* European colonization events, while red track represents ancient North American colonization route *via* Beringia. Presumed origins of related *Humulus* species are highly speculative. **(B)** Approximate locations for 30 wild-growing Maritimes hops accessions analyzed in this study. Site names and exact locations deliberately obscured to protect landowners. For further details, see **Table 2** and body of text.

The global hops trade is worth approximately $500 million (US) annually, with commercial scale production spanning North America, Europe, East Asia, and Australia/New Zealand (FAOSTAT, 2019). Hops (*Humulus lupulus* L.), a member of the family *Cannabaceae*, is a hardy climbing perennial plant native to the northern hemisphere (**Figure 1A**). It has a dioecious reproductive strategy and flowers are wind-pollinated. Female hops flowers, also known as cones, are primarily used in brewing beer; most of the bitter flavor and characteristic zesty beer aromas arise from hops cones added at various points during wort boiling, secondary fermentation, and the aging process. A wide variety of phytochemicals, some uniquely found in hops, have previously been reported (Almaguer et al., 2014; Dresel et al., 2016; Karabín et al., 2016), with a particular focus on prenylflavonoids, alpha and beta acids (soft resins), and volatile terpenoids. In addition to their importance in beer brewing, hops have been used in traditional herbal medicines as a sleep aid, while modern biomedical research has focused on various components in the development of new therapeutic agents (Stevens and Page 2004; Gerhäuser 2005; Zanoli and Zavatti 2008; Van Cleemput et al., 2009; Karabín et al., 2016).

**Figure 2:**
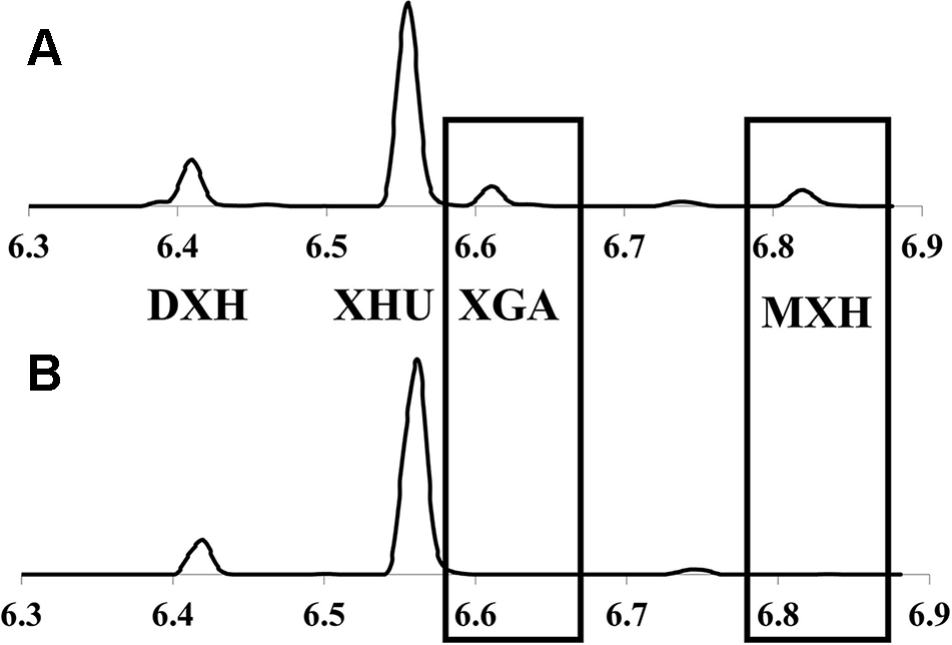
Representative UPLC-DAD chromatograms (370nm) of hops cone prenylchalcones showing: **(A)** *lupuloides*-chemotype profile possessing xanthogalenol (XGA) and 4’-O-methyl xanthohumol (MXH); and **(B)** *lupulus*-chemotype profile lacking these chemotaxonomic markers. XHU, xanthohumol; DXH, desmethylxanthohumol.

Taxonomic classification and delineation of hops at the species and/or subspecies level has undergone continuous debate and revision, with the utilization of molecular phylogenetics (Murakami et al., 2006; Bassil et al., 2008; Townsend and Henning, 2009; Patzak et al., 2010b), physiological/morphological characteristics (Small, 1978; Tembrock et al., 2016), chemotaxonometric (Stevens et al., 2000; Hampton et al., 2002; Hummer et al., 2005; Vašek et al., 2013), and paleobotany findings (MacGinitie, 1953; Manchester, 2001) being implemented. There is a general consensus that North American and European hop lineages are separated by more than a million years of evolution from their last common ancestor, likely diverging somewhere in the Altai region of Central Asia (Murakami et al., 2006); these distinct lineages are readily distinguished by molecular and chemical markers, with two prenylchalcone derivatives, xanthogalenol (XGA) and 4’-*O*-methyl xanthohumol (MXH) (**Figure 2**) being entirely absent in European hops (Stevens et al., 2000; Hampton et al., 2002).

Within North America, three native subspecies (*H. lupulus* ssp. *neomexicanus*; *H. lupulus* ssp. *pubescens*; and *H. lupulus* ssp. *lupuloides*) have previously been defined (**Figure 1A**), based on a combination of morphological and chemometric markers (Small, 1978; Stevens et al., 2000; Hampton et al., 2002), although recent analysis suggests their potential reclassification as separate and individual species (Tembrock et al., 2016). Controversial fossil evidence from the Florissant beds in Colorado suggests hops may have been present in North America 33 million years ago (MacGinitie, 1953; Manchester, 2001), however, the greater diversity of *Humulus* species present in Asia along with geographic proximity to the related genus *Cannabis* suggests ancient Asian or Laurasian origins for hops. The presumed route for North American colonization is a coastal migration *via* the now submerged region of Beringia, with subspecies diverging approximately 500,000 years ago (Murakami et al., 2006). This hypothesis is further supported by shared chemical, morphological, and molecular similarities between North American and Japanese hops (*H. lupulus* ssp. *cordifolius*), distinguishing both groups from European lineages. Of note, the modern day, northernmost distribution limit of *lupuloides* exceeds the southernmost extent of the Laurentide icesheet at glacial maxima 21,500 years ago, indicating widespread recolonization of these latitudes from cryptic Pleistocene refugia areas (Soltis et al., 2006), perhaps occurring multiple times during interglacial cycles. The impact of the Appalachian Mountains in shaping recolonization and disrupting gene flow between western and eastern *lupuloides* populations remains speculative.

Through the 17th to 19th centuries, British, Dutch, and French colonists brought European hop (*Humulus lupulus* ssp. *lupulus*) varieties/landraces with them to the new world, but they were poorly suited to the cool, wet, and humid climates of eastern North America. These populations were especially susceptible to insect and disease pressures (Tomlan, 1992). Early settlers used native hops (*lupuloides*) for brewing purposes, along with spontaneous hybrids arising from open pollination of cultivated European stocks, eventually resulting in the development of naturalized *lupulus* cultivars such as “Cluster”, widely regarded amongst the oldest American hop varieties. The frequency of spontaneous hybridization events between native *lupuloides* and introduced *lupulus* has been reported to be quite low, although intentional crosses yield fertile offspring (Tembrock et al., 2016). Commercial hop production in the late 19th and early 20th centuries continuously migrated westwards in response to declining yields and disease pressures including a newly emergent pathogen (Downy Mildew - *Pseudoperonospora humuli*) (Tomlan, 1992). The majority of North American hop production eventually became centralized in the arid mountain valleys of the Pacific North West (PNW), specifically the American states of Washington, Oregon, and Idaho (**Table 1**). This area is still the predominant hops producing region of the world producing more than half of the globe’s total supply (Small, 2016; FAOSTAT, 2019). Over the past five decades, hops breeding programs have developed numerous commercial varieties well-suited to the hot, dry, mountain valleys of the PNW region.

**Table 1:** Thirty-year climatic data norms (1970–2000) for Yakima Washington, Charlottetown Prince Edward Island, and Morden Manitoba, representative of distinct growing regions for commercially produced and wild *lupuloides* germplasm resources.

Location	*Yakima, Washington, USA*					GDD	2200	HZ	6b/7a	FFD	160	
**Month**	**JAN**	**FEB**	**MAR**	**APR**	**MAY**	**JUN**	**JUL**	**AUG**	**SEP**	**OCT**	**NOV**	**DEC**
Avg Temp (°C)	–1.5	2.2	5.9	9.6	14.0	18.0	21.2	20.7	16.1	9.7	3.4	–1.1
Min Temp (°C)	–5.9	–3.2	–0.1	2.0	5.9	9.8	12.0	11.4	7.1	1.7	–1.9	–5.2
Max Temp (°C)	2.9	7.6	12.8	17.3	22.2	26.3	30.5	30.0	25.2	17.8	8.8	3.1
Precipitation (mm)	31	20	17	13	13	15	5	9	10	14	27	33
**Location**	**Charlottetown, Prince Edward Island, Canada**					**GDD**	**1650**	**HZ**	**5b**	**FFD**	**160**	
**Month**	**JAN**	**FEB**	**MAR**	**APR**	**MAY**	**JUN**	**JUL**	**AUG**	**SEP**	**OCT**	**NOV**	**DEC**
Avg Temp (°C)	-6.8	–6.8	–2.6	3.1	9.1	14.6	18.7	18.1	13.8	8.4	3.2	–3.4
Min Temp (°C)	–10.8	–11.0	–6.4	–0.8	4.2	9.6	13.9	13.4	9.2	4.3	0.0	–6.9
Max Temp (°C)	–2.7	–2.6	1.3	7.1	14.1	19.7	23.6	22.9	18.5	12.5	6.5	0.2
Precipitation (mm)	106	86	86	84	85	83	81	91	86	103	117	119
**Location**	**Morden, Manitoba, Canada**					**GDD**	**1800**	**HZ**	**4a**	**FFD**	**130**	
**Month**	**JAN**	**FEB**	**MAR**	**APR**	**MAY**	**JUN**	**JUL**	**AUG**	**SEP**	**OCT**	**NOV**	**DEC**
Avg Temp (°C)	–16.7	–12.9	–6.1	4.1	11.9	17.2	20.0	18.8	12.8	6.6	–3.9	–12.6
Min Temp (°C)	–21.6	–18.2	–11.3	–1.9	4.8	10.7	13.6	12.1	6.6	0.9	–8.2	–17.2
Max Temp (°C)	–11.7	–7.6	–0.9	10.2	19.1	23.8	26.5	25.5	19.1	12.3	0.4	–8.0
Precipitation (mm)	20	16	24	37	61	78	73	67	50	33	22	19

GDD, cumulative growing degree days above 5°C April 1- Oct 31, 1971-2000 30 year average.

HZ, USDA hardiness zone.

FFD, average number frost free days.

Due to emerging disease pressures in Britain and Europe, early hops breeding efforts (1910–1930) at Wye College (Wye, Kent, UK) by Ernest Salmon endeavored to develop pathogen resistance traits through introgression from *lupuloides* germplasm resources, with wild plants collected by William Macoun in 1916 at Morden, Manitoba, Canada (**Table 1**). Progeny from these crosses include famous named cultivars such as “Brewer’s Gold” and “Northern Brewer”, themselves strongly contributing to the pedigrees of the majority of today’s most commercially important American hops cultivars. While *lupuloides* genetic resources from the Great Plains regions of North America (Saskatchewan, Manitoba, Montana, North Dakota) have continued to be evaluated for potential use in hops breeding and cultivar development (Stevens et al., 2000; Hampton et al., 2001; Hampton et al., 2002; Hummer et al., 2005; Bassil et al., 2008), to our knowledge, the diversity and characteristics of eastern *lupuloides* germplasm resources are completely unknown.

Being geographically isolated from the main *lupuloides* population in central North America by the Appalachian Mountains and undergoing evolutionary selection in a wetter, cooler climate (**Table 1**), these Maritimes region *lupuloides* populations can be expected to possess significantly different chemical profiles, pest-resistance, and phenological traits than those previously characterized from the northern Great Plains. Assuming a Beringian colonization route for North America by an ancient hops precursor (**Figure 1A**), *lupuloides* individuals found in the Maritimes region of Canada represent the furthest geographical distance travelled away from the origin of species in central Asia. Numerous opportunities for genetic bottlenecks and population founder effects, in part due to recolonization of northern latitudes from Pleistocene refugia areas following glaciation cycles, increases the potential for interesting phenotypic uniqueness within this Maritimes *lupuloides* population.

Recent growth of the craft brewing industry across North America, tied in part to the local foods movement and gastrotourism, has created demand for locally-produced and regionally-distinct hops varieties. In general, the craft beer industry actively embraces novelty in flavors and aromas, thereby promoting demand for new hops varieties. Unfortunately, most existing cultivars developed for the PNW region over the past five decades perform quite poorly in the eastern regions of North America; cultivars bred, selected, and evaluated under dry growing seasons in arid mountain valleys suffer from a variety of agronomic performance issues when planted in New England and the Canadian Maritimes regions. The climate of Charlottetown, PE, Canada, is representative of the Canadian Maritimes region, being characterized by a cool, wet growing season (April 1–October 31) (**Table 1**). Compared to the Yakima valley in Washington, the epicenter of North American hops production, the Maritimes region of Canada has a much cooler growing season (∼550 fewer growing degree days above 5°C), while also accumulating nearly 8 times as much precipitation; dramatic differences in plant performance and pest pressures exist between these regions. The northern Great Plains region, as exemplified by Morden, Manitoba (**Table 1**), itself being the source of critical *lupuloides* germplasm resources utilized in the past century, has a much colder winter, shorter but warmer growing season and less precipitation than the Maritimes region, and in some ways is more similar to the PNW. Developing new hops cultivars well-suited to the cooler, wetter eastern half of the continent is critical to supporting sustained growth of the craft-brewing industry. In support of these activities, we have assembled a diverse collection of wild-growing Maritimes hops plants, including both *lupuloides* and *lupulus* accessions, to assess their agronomic potential and chemical diversity. Phytochemical characterization of this collection, including leaf and cone chemical profiles was quantitated and is reported herein.

## Results and Discussion

A total of 61 wild-growing hops populations were identified and visited, spread across the three Canadian Maritime provinces, located between 44°1’ to 46°6’N and 67°5’ to 60°3’W, and spanning an area of roughly 75,000 km^2^. A variety of habitats were sampled, including abandoned farmsteads, interval land, and saltwater estuaries, heavily forested natural areas, and along roadsides (**Table 2**). Rhizomes were collected from these sites for further plant propagation and centralized maintenance of germplasm resources within Agriculture and Agri-Food Canada (AAFC) at Harrington Research Farm in Prince Edward Island, Canada. Of these 61 accessions, 30 sites yielded cones during the period of study (**Figure 1B**), for further chemical and taxonomic analysis.

**Table 2:** Combined chemical traits (prenylchalcone diversity, alpha acid characteristics, beta acid content, acylated flavonol composition) for 30 wild-collected maritimes hops accessions including native *lupuloides* and feral *lupulus* lineages.

Site Code	Subspecies Identification	General Habitat	Total			XGA	MXH	Total			Proportion COH	Total			Alpha ratio			Proportion Mal-Fla (Ka + Qc)		SE
			prenylchalcones					alpha acids				beta acids								
			(% dw)		SE	(% dw)	(% dw)	(% dw)		SE	(COH/Σaa)	(% dw)		SE						
S-JS	*lupuloides*	farmstead	0.47	±	0.02	0.036	0.030	5.67	±	0.16	0.40	3.97	±	0.09	0.59			0.055		
B-GC	*lupuloides*	forested	0.59	±	0.03	0.041	0.044	6.10	±	0.42	0.47	4.41	±	0.26	0.58			0.022		
S-BA	*lupuloides*	farmstead	0.62	±	0.03	0.049	0.040	6.29	±	0.23	0.40	5.37	±	0.16	0.54			0.021		
P-TG	*lupuloides*	farmstead	0.48	±	0.02	0.030	0.025	5.68	±	0.27	0.41	5.46	±	0.25	0.51			0.063		
P-CB	*lupuloides*	fresh water marsh	0.58	±	0.01	0.038	0.033	6.28	±	0.17	0.41	5.58	±	0.12	0.53			0.028		
P-CC	*lupuloides*	estuary	0.63	±	0.02	0.042	0.042	6.96	±	0.22	0.42	5.65	±	0.18	0.55			0.029		
S-MD	*lupuloides*	farmstead	0.53	±	0.01	0.034	0.031	6.16	±	0.30	0.41	5.68	±	0.11	0.52			0.031		
P-WR	*lupuloides*	riparian	0.74	±	0.04	0.048	0.045	7.74	±	0.51	0.42	5.72	±	0.39	0.58			0.058		
P-CP	*lupuloides*	fresh water marsh	0.65	±	0.05	0.043	0.039	7.25	±	0.47	0.42	5.74	±	0.40	0.56			0.044		
P-DU	*lupuloides*	forested	0.62	±	0.03	0.036	0.036	6.75	±	0.37	0.42	5.75	±	0.24	0.54			0.027		
P-MV	*lupuloides*	riparian	0.54	±	0.03	0.046	0.040	6.22	±	0.36	0.40	5.82	±	0.21	0.52			0.028		
P-MR	*lupuloides*	riparian	0.74	±	0.04	0.052	0.047	7.84	±	0.42	0.41	6.02	±	0.18	0.57			0.036		
S-TR	*lupuloides*	farmstead	0.62	±	0.01	0.041	0.038	7.34	±	0.16	0.39	6.03	±	0.18	0.55			0.030		
S-SC	*lupuloides*	roadside	0.72	±	0.04	0.048	0.045	7.96	±	0.32	0.39	6.30	±	0.27	0.56			0.028		
B-TW	*lupuloides*	farmstead	0.81	±	0.03	0.062	0.057	9.38	±	0.18	0.40	6.34	±	0.12	0.60			0.026		
P-WG	*lupuloides*	roadside	0.71	±	0.03	0.049	0.042	7.68	±	0.26	0.42	6.47	±	0.16	0.54			0.036		
B-SG	*lupuloides*	estuary	0.69	±	0.03	0.040	0.041	8.99	±	0.32	0.40	6.98	±	0.26	0.56			0.035		
B-FG	*lupuloides*	farmstead	0.88	±	0.02	0.098	0.074	2.53	±	0.05	0.39	9.93	±	0.19	0.20			0.028		
S-AP	*lupuloides*	estuary	1.15	±	0.07	0.111	0.087	3.51	±	0.14	0.36	12.0	±	0.64	0.23			0.027		
P-NW	*lupulus*	farmstead	0.52	±	0.02	0.000	0.000	6.07	±	0.23	0.26	2.12	±	0.09	0.74			0.324		
P-HF	*lupulus*	old hopyard	0.58	±	0.01	0.000	0.000	5.95	±	0.17	0.32	2.55	±	0.04	0.70			0.221		
B-DX	*lupulus*	farmstead	0.65	±	0.03	0.000	0.000	8.31	±	0.44	0.31	2.70	±	0.13	0.75			0.531		
P-HR	*lupulus*	riparian	0.51	±	0.01	0.000	0.000	5.32	±	0.22	0.29	2.74	±	0.07	0.66			0.386		
P-WB	*lupulus*	farmstead	0.60	±	0.03	0.000	0.000	5.21	±	0.35	0.33	3.85	±	0.20	0.57			0.511		
S-MI	*lupulus*	riparian	0.29	±	0.03	0.000	0.000	1.70	±	0.13	0.19	4.15	±	0.37	0.29			0.388		
S-89	*lupulus*	farmstead	0.38	±	0.02	0.000	0.000	2.16	±	0.10	0.18	4.98	±	0.24	0.30			0.383		
P-WK	*lupulus*	farmstead	0.64	±	0.03	0.000	0.000	7.33	±	0.70	0.36	5.50	±	0.37	0.57			0.242		
S-WM	*lupulus*	riparian	0.44	±	0.03	0.000	0.000	2.33	±	0.13	0.21	6.29	±	0.49	0.27			0.290		
S-11	*lupulus*	roadside	0.51	±	0.03	0.000	0.000	2.94	±	0.16	0.18	6.54	±	0.35	0.31			0.536		
S-81	*lupulus*	roadside	0.50	±	0.03	0.000	0.000	3.05	±	0.15	0.17	6.55	±	0.27	0.32			0.491		
	**avg** ***lupuloides***		0.67		0.04	0.050	0.044	6.65	±	0.38	0.41	6.27	±	0.42	0.52	±	0.03	0.034	±	0.003
	**avg** ***lupulus***		0.51	±	0.03	0.000	0.000	4.58	±	0.68	0.26	4.36	±	0.51	0.50	±	0.06	0.391	±	0.035
	**avg entire collection**		0.61	±	0.03			5.89	±	0.39	0.35	5.57	±	0.36	0.51	±	0.03	0.165	±	0.034

S, Nova Scotia; B, New Brunswick; P, Prince Edward Island. Dw, dry weight; XGA, xanthogalenol; MXH, 4’-O-methyl xanthohumol; COH, cohumulone; ∑aa, total alpha acids; Mal-Fla, malonylated flavonols; Ka, kaempferol; Qc, quercetin; SE, standard error of mean.

### Prenylchalcone Quantitation and Diversity

Prenylchalcone diversity has been previously demonstrated to be a reliable chemotaxonomic marker of hops ancestry, with North American lineages (specifically *lupuloides*) producing XGA and MXH as useful taxonomic markers (Stevens et al., 2000; Hampton et al., 2002). Prenylchalcone profiling of hops cones within our collection by UPLC-DAD-MS/MS (**Figure 2**) revealed a diversity in XGA and MXH chemotypes (**Table 2**), indicating our germplasm repository contains both native North American *H. lupulus* ssp. *lupuloides* (63.3%) and feral European *H. lupulus* ssp. *lupulus* (36.7%) accessions, consistent with previous reports based on numeric morphological analysis of herbarium specimens from the region (Small, 1978). Feral *lupulus* accessions tended to be found in proximity to populated areas and old farmsteads and were often associated with apple trees, while *lupuloides* accessions were generally found in close proximity to riparian and forested areas and interval land between the two, which is consistent with what has previously been reported (Hampton et al., 2001) (**Table 2**).

Xanthohumol (**XHU**) was the major prenylchalcone present in all samples followed by desmethylxanthohumol (**DXH**), as previously reported by Stevens et al. (1999; 2000). The levels of total prenylchalcones ranged from 0.29% ± 0.03% to 1.15% ± 0.07%, by dry weight (dw), comparable to commercial cultivars produced in the region and quantitated using the same analytical techniques (0.32%–0.85% dw; data not shown). Individuals showing *lupulus* chemotypes tended toward the lower end of this range (x̅ = 0.51%), while *lupuloides* chemotypes were generally higher (x̅ = 0.67%), but these differences were not found to be entirely predictive of ancestry (**Table 2**). *Lupuloides* chemotype individuals displayed a XGA content ranging from 0.0295% to 0.111% dw, while MXH ranged from 0.0254% to 0.0868% dw, with XGA being generally more abundant than MXH.

The levels of prenylchalcones for these Maritimes region *lupuloides* accessions are ∼2–3 times higher than those previously reported for western *lupuloides* germplasm resources by Patzak et al., 2010b (Canada x̅ = 0.22%; USA x̅ = 0.38%) and by Hampton et al., 2002 (x̅ = 0.17%), and are comparable to previous reports from commercially-sourced hops cones and pellets which typically contain around 0.5% xanthohumol by dw (Stevens, 1967; De Keukeleire et al., 2003; Stevens and Page, 2004; De Keukeleire et al., 2007; Magalhães et al., 2007; Čeh et al., 2007). It is unclear how environmental factors (light quality, temperature, precipitation, pest pressures, agronomic management) during hops cone development, differences in individual extraction methodologies, source materials (whole cones versus processed hops pellets), age of samples (fresh vs. commercially sourced), and plant genetics combine to explain these results. Prenylchalcones arise from generalized flavonoid biosynthesis, with many of the early stage enzymes (PAL, C4H, 4CL, CHS) being highly inducible by pathogen attack and environmental stresses (Gould and Lister, 2006), with previous studies showing poor weather conditions and organic farming practices resulting in higher levels of xanthohumol accumulation (De Keukeleire et al., 2007). Xanthohumol and its derivatives have been shown to possess *in vitro* antifungal effects (Gerhäuser, 2005; Bartmańska et al., 2018), and likely function similarly *in planta*, being synthesized, stored, and subsequently released by peltate glandular trichomes present on the leaf abaxial surfaces, in close proximity to stomatal openings targeted by opportunistic plant pathogens (Roye and Thomas, 1971). Higher disease pressures experienced in Maritimes Canada as compared to other growing regions may partially explain these elevated results.

### Alpha and Beta Acid Quantitation and Diversity

**Figure 3:**
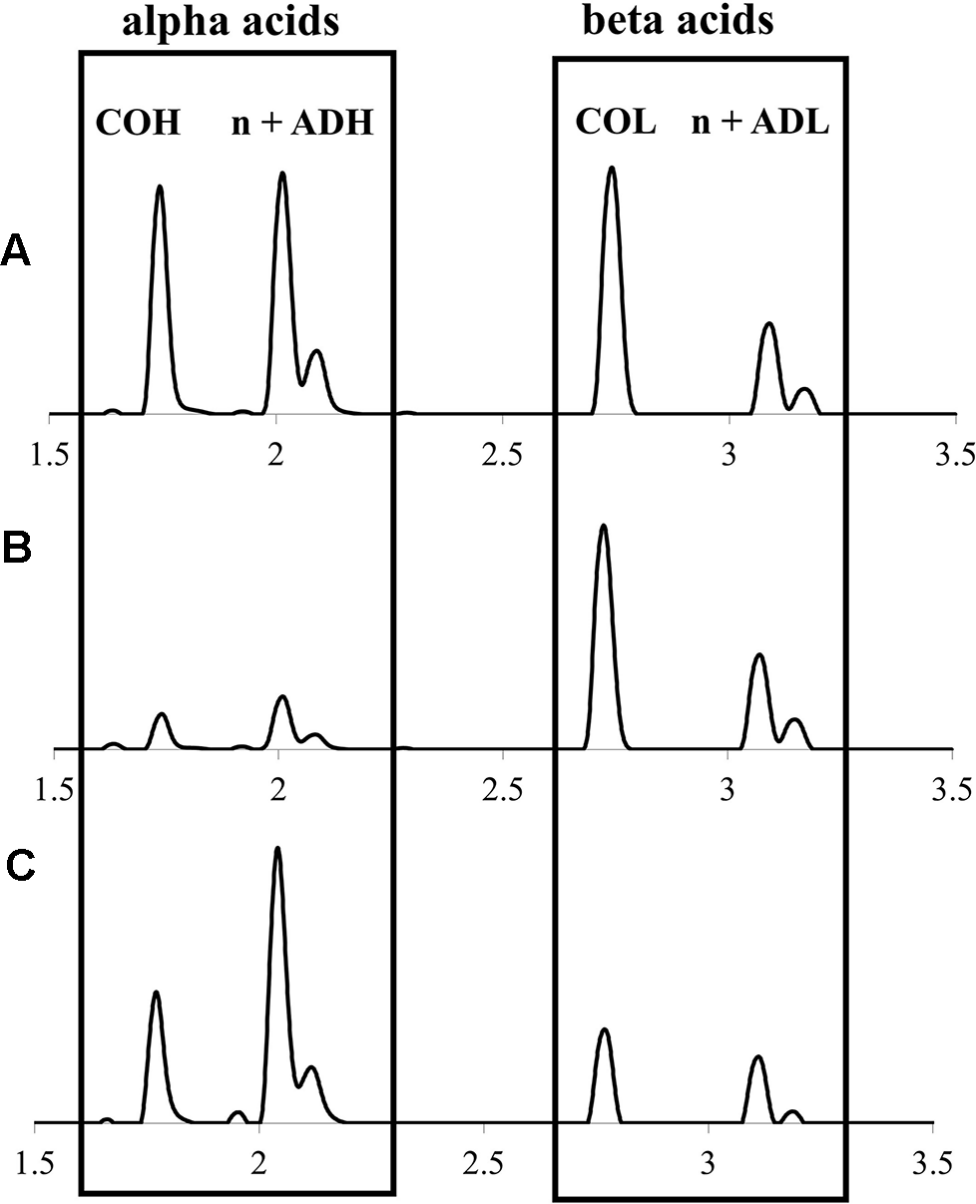
Representative UPLC-DAD chromatograms (320 nm) of hops cone soft resin diversity (alpha and beta acids) showing: **(A)** *lupuloides* high alpha acid chemotype profile; **(B)** *lupuloides* high beta acid chemotype profile; **(C)** *lupulus* low co-humulone chemotype profile. COH, cohumulone; n + ADH, humulone + adhumulone; COL, colupulone; n + ADL, lupulone + adlupulone. Note n + ad isomers have traditionally been reported as one peak, due to past difficulties in their chromatographic resolution.

Alpha and beta acids from hops cones were quantitatively profiled by UPLC-DAD-MS/MS (**Figure 3**), revealing a diversity in both the total quantities and ratios of individual components (**Table 2**). Total alpha acids, of critical importance to the brewing industry due to their bittering effects, were found to range from 1.70% ± 0.13% to 9.38% ± 0.18% dw (**Table 2**), comparable to commercial cultivars (2.75%–14.6%) produced in the region under similar environmental and agronomic conditions (data not shown). Feral Maritimes region *lupulus* accessions clustered toward the lower end of this range (x̅ = 4.58%), while also generally displaying a lower proportion of cohumulone (x̅ = 0.26), which is considered to be a defining trait typical of European hops varieties (Patzak et al., 2010a). Maritimes region *lupuloides* accessions tended to produce higher levels of alpha acids (x̅ = 6.65% dw) and display higher cohumulone proportions (x̅ = 0.41), a characteristic of native North American hops (Hampton et al., 2002; Patzak et al., 2010b). Similar to total prenylchalcone content (see Prenylchalcone Quantitation and Diversity above), *lupuloides* accessions from the Maritimes region display higher total alpha acid values (x̅ = 6.65% dw) than those previously reported from the westernmost extent of their range by Patzak et al., 2010b (Canada x̅ = 2.42%; USA x̅ = 3.60%) and by Hampton et al., 2002 (x̅ = 3.80%). Interestingly, the cohumulone (COH) proportion in Maritimes *lupuloides* accessions, a ratio of cohumulone content per total alpha acids, was much lower (x̅ = 0.41) compared to Great Plains *lupuloides*, as previously reported by Patzak et al., 2010b (Canada x̅ = 0.55; USA x̅ = 0.57) and Hampton et al., 2002 (x¯ = 0.58).

While not comparable to commercially released “super alpha” cultivars, which include hops developed primarily for bittering purposes and containing 15%–20% or higher total alpha acids, the intermediate levels of alpha acids (6%–9% dw) characteristic of individual accessions we collected, show potential use as versatile, dual-purpose (aroma and bittering) varieties. While the cohumulone ratios in Maritimes region *lupuloides* germplasm materials are higher than many commercial cultivars of European origins, they are much lower than previously reported *lupuloides* germplasm materials (Hampton et al., 2002; Patzak et al., 2010b). Modern brewing methods and recipe optimization easily accommodate for the cohumulone levels observed in Maritimes accessions. Due to its increased polarity and lower pKa value, cohumulone is more soluble than other alpha acids while also undergoing more efficient isomerization during wort boiling (Jaskula et al., 2008). As a result, hops with high proportions of cohumulone produce more bittering effect for a given alpha acid content. Without adjusting for cohumulone levels, inexperienced brewers can produce an unintentionally bitter beer, which has historically cast wild North American hops in a negative light. Many popular commercial cultivars, such as “Cascade”, “Cluster”, “Newport”, “Columbia” and “Galena” contain cohumulone proportions (∼0.39–0.41) similar to those in our collection (USDA, 2007).

In general, beta acids have been ignored by the brewing industry, being essentially insoluble in wort and nearly absent from finished beer products, but they may play a vital role in invertebrate pest deterrence (Naraine and Small, 2017), and in the development of natural health products and other therapeutic agents, being strongly active against Gram-positive bacteria (Schmalreck et al., 1975) and protozoa (Srinivasan et al., 2004), while possessing anti-cancer and anti-inflammatory activities (Zhao et al., 2005; Zanoli and Zavatti 2008; Van Cleemput et al., 2009).

Maritimes hops accessions displayed total beta acids ranging from 2.12% ± 0.09% to 12.0% ± 0.64% dw (**Table 2**), comparable to commercial cultivars (1.21%–9.35%) produced in the region under similar environmental and agronomic conditions (data not shown). Maritimes *lupulus* accessions were found to cluster toward the lower end of this range (x̅ = 4.36%), while *lupuloides* accessions were at the higher end (x̅ = 6.27%), but these results were not definitively predictive of ancestry. Maritimes *lupuloides* accessions were found to possess higher beta acid levels than those previously reported from the westernmost extreme of their range by Patzak et al., 2010b (Canada x̅ = 3.57%; USA x̅ = 3.93%) and by Hampton et al., 2002 (x¯ = 3.24%).

The proportion of alpha acids, Pα, defined as the amount of alpha acids divided by the total content of alpha and beta acids was found to be 0.50 for Maritimes region *lupulus* accessions and 0.52 for *lupuloides* accessions. Both these values are lower than commercial cultivars grown in the region (Pα = 0.61), produced under similar climatic and agronomic practices (data not shown), but are comparable to previous reports of *lupuloides* from the westernmost extreme of their range by Patzak et al., 2010b (Canada Pα = 0.40; USA Pα = 0.47) and by Hampton et al., 2002 (Pα = 0.54). This balanced proportion of alpha to beta acids in *lupuloides* is apparently consistent across the entire population range; however, the total amount of bitter acids is markedly higher in the Maritimes. Certain individuals within our germplasm collection, (B-FG, S-AP), were found to possess very high levels and proportions of beta acids (**Table 2**), and maybe of further use in developing hops cultivars for natural health products and therapeutics applications focusing on lupulone derivatives. Molecular characterization of polymorphisms in key biosynthetic enzymes influencing the distribution of bitter acid in these individuals is in progress.

### Leaf Flavonol Diversity

A thorough investigation of the phytochemical content of hops leaves was conducted, as this is the principal location for colonization and invasion by pathogenic microorganisms (Roye and Thomas, 1971) and feeding damage caused by invertebrate herbivores (Naraine and Small, 2017). While abaxial peltate glandular trichomes have been previously reported to produce a mixture of prenylchalcones, beta acids and terpene derivatives (Sugiyama et al., 2006; Wang et al., 2008; Patzak et al., 2015), to our knowledge, the chemical composition of vegetative photosynthetic tissues from hops leaves has not been previously reported.

**Figure 4:**
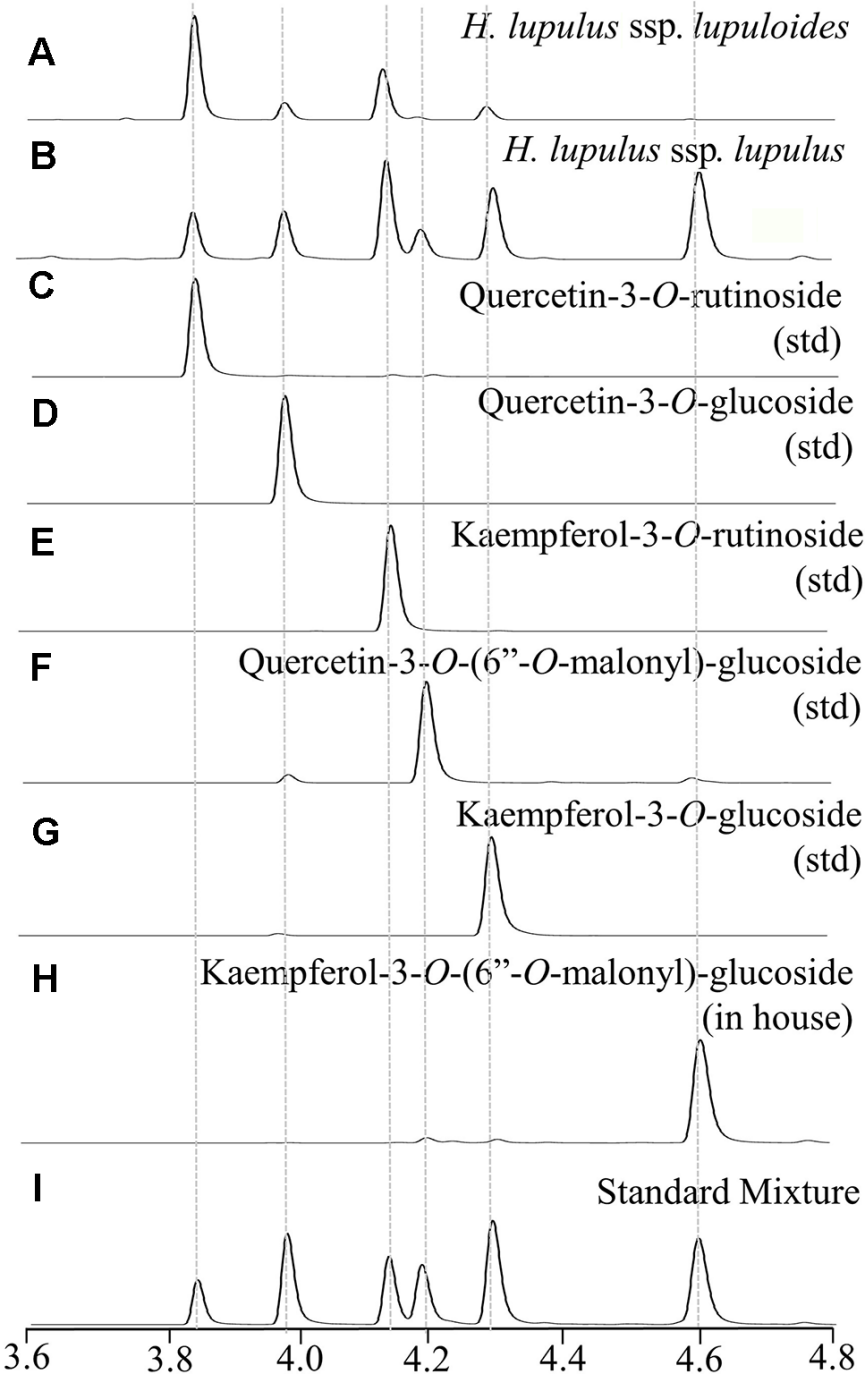
Representative UPLC-DAD chromatograms (350 nm) displaying hops leaf flavonol diversity showing: **(A)** *lupuloides* low malonyl ester chemotype profile; **(B)** *lupulus* high malonyl ester chemotype profile; **(C**–**G)** commercially sourced analytical standards (std); **(H)** in house purified peak; **(I)** reconstructed profile using mixture of standards.

Hops leaves were found to contain a variety of flavonol glycosides comprised of both quercetin and kaempferol derivatives (**Figure 4**, **Table 3**), ranging in total concentration from 0.28% to 2.77% dw (quercetin-3-*O*-rutinoside equivalents), although the total values were highly dependent on phenological development stage, sampling date, and physical location along the bine (data not shown). The identity of individual flavonol constituents was unequivocally validated through a combination of UV-Vis absorption spectra, MS/MS analysis, congruent retention time with authentic standards, and spiking experiments (**Figures 4C–G**, **Table 3**).

**Table 3:** Chromatographic, UV-vis spectroscopic, and Mass Spectrometric characteristics of Hops leaf Flavonol compounds.

Flavonol glycoside peak	RT (min)	UV-Vis (λmax) (nm)	ESI+ve ions (abundance)	ESI-ve ions (abundance)
quercetin-3-O-rutinoside	3.83	354	611.4 (100) [M+H]^+^ ; 303.1 (1) [aglycone + H ]^+^	609.3 (100) [M-H]^-^ ; 1219.3 (30) [2M-H]^-^
quecetin-3-O-glucoside	3.96	354	465.3 (100) [M+H]^+^ ; 303.0 (8) [aglycone + H ]^+^	463.3 (100) [M-H]^-^ ; 927.3 (60) [2M-H]^-^
kaempferol-3-O-rutinoside	4.18	347	595.3 (100) [M+H]^+^ ; 287.7 (2) [aglycone + H ]^+^	593.3 (100) [M-H]^-^ ; 1187.6 (20) [2M-H]^-^
quercetin-3-O-(6''-O-malonyl)-glucoside	4.20	354	551.3 (100) [M+H]^+^	505.3 (90) [M-CO2]^-^ ; 549.2 (25) [M -H]^-^ ; 1099.6 (100) [2M-H]^-^
kaempferol-3-O-glucoside	4.30	348	449.3 (100) [M+H]^+^ ; 287.4 (8) [aglycone + H ]^+^	447.4 (100) [M-H]^-^ ; 895.4 (35) [2M-H]^-^
kaempferol-3-O-(6''-O-malonyl)-glucoside	4.60	348	535.2 (100) [M+H]^+^	489.3 (100) [M-CO2]^-^ ; 533.2 (35) [M -H]^-^ ; 1067.4 (95) [2M-H]^-^

**Figure 5:**
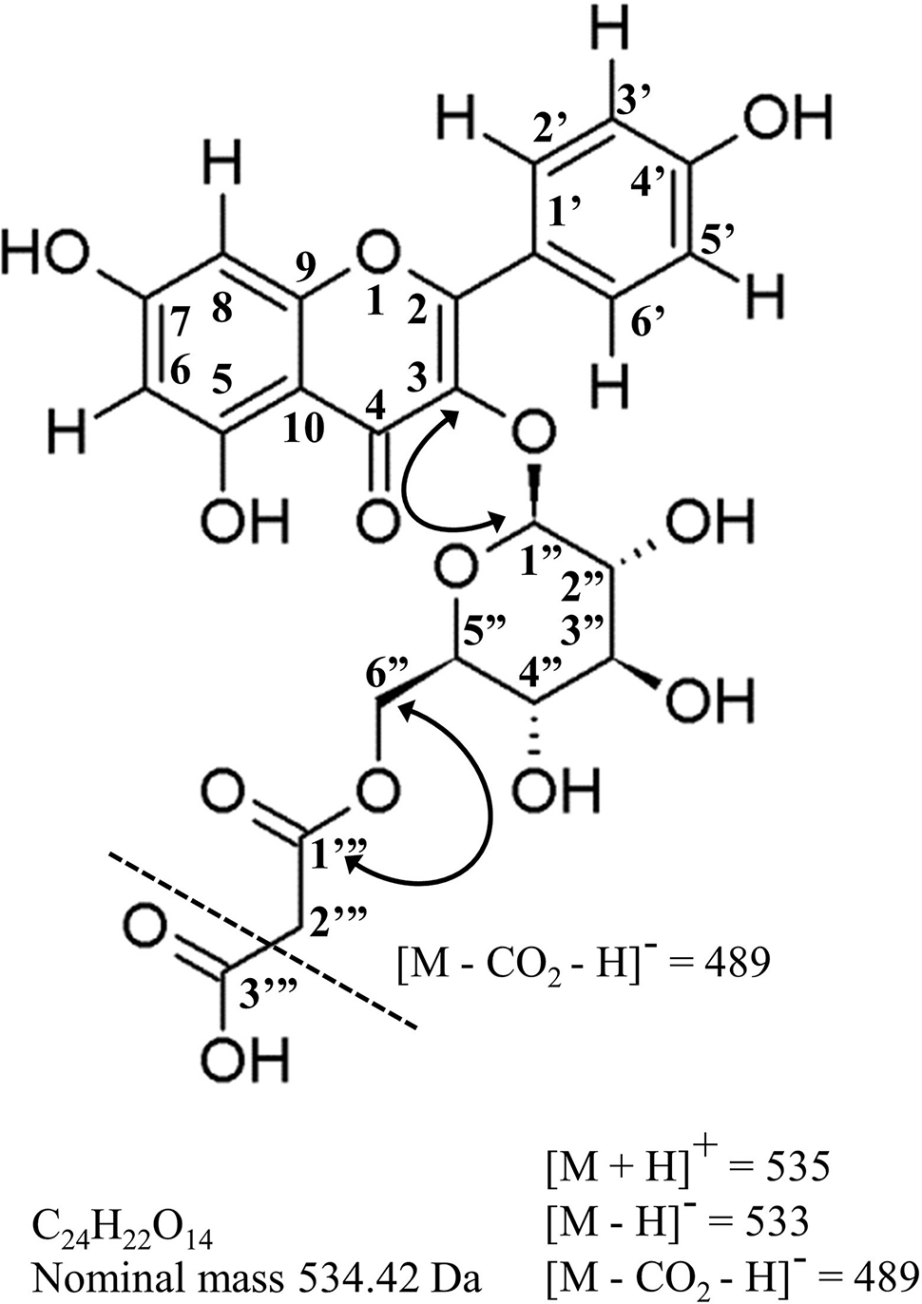
Molecular structure, mass-spectral characteristics and important NMR correlations used to identify kaempterol-3-*O*-(6’’-*O*-malonyl)-β-D-glucopyranoside, the principal acylated flavonol in hops leaves, and a potential new chemotaxonomic marker of hops ancestry. Standard flavonoid numbering system used herein and in **Table 4**. Solid arrowed line = important HMBC correlations; dashed line = principle ion in ESI- (loss of terminal carboxy group); Da = daltons.

Two distinct chemotypes were observed within our collection; a profile rich (> 0.20 +; **Figure 4B**) or depleted (<0.10; **Figure 4A**) in the proportion of total malonyl esters of kaempferol and quercetin-3-*O*-glucosides, although trace levels of these acylated flavonols were still identifiable in every sample by MS-MS analysis, generating ESI- quasimolecular ions and fragments of 533/489 and 549/505, respectively (**Figure 5**, **Table 3**). For most accessions, the kaempferol derivative was the principal malonylated compound, being about five times more abundant than the quercetin analogue. In addition to its greater overall abundance, the kaempferol derivative is better resolved chromatographically than its quercetin analogue, making it a more attractive chemical marker. Intriguingly, of the Maritimes accessions identified as *lupuloides* due to the presence of XGA and MXH (see Prenylchalcone Quantitation and Diversity above), all possessed the low malonyl ester chemotype (**Figure 4A**), averaging only 0.0343 by relative abundance (**Table 2**); those identified as *lupulus* and lacking XGA and MXH peaks all possessed the high malonyl ester chemotype (**Figure 4B**), averaging 0.391 by relative abundance (**Table 2**). The association between low flavonoid malonyl ester content in leaves and XGA/MXH positive chemotypes in cones is 100%, for the 30 hops accessions where we have complete phytochemical data.

We believe the low malonyl ester chemotype may be useful in further delineation/identification of *lupuloides* versus *lupulus* individuals, both within our germplasm collection, along with archived herbarium specimens and for future botanical surveys; particularly for individuals currently lacking cone prenylchalcone chemotype data. Leaf sampling from wild-growing hops plants is a far simpler task than collecting cones, which are located up to 10 meters off the ground in heavily wooded and overgrown areas, and are only present late in the growing season. Additionally, as male hops plants do not generate cones, leaf-based chemotyping based on flavonol profiles may allow for facile classification of these individuals, as opposed to relying on DNA-based or microscopy techniques.

It is unclear how the strong correlation observed between low flavonol malonyl ester content in leaves and XGA/MXH methylation profile of cone prenylchalcones arises; these characteristics are expected to result from distinct genetic loci with separate flavonoid-glycoside-malonyltransferase and flavonoid-*O*-methyl transferase enzyme activities. Whether alleles for these traits have become fixed in eastern North American versus European populations as a result of genetic drift or other selection pressures is a potential future area of study. As existing hops genome studies are still in draft assembly forms (Natsume et al., 2015; Hill et al., 2017), we are currently unable to determine whether the putative *O*-methyltransferase and flavonoid-malonyltransferase genes underlying these inversely correlated traits are tightly linked on a chromosome; further chemotyping of hybrid commercial cultivars with known pedigrees may shed some light on the matter of segregation, dominance and penetrance of these traits.

### Identification of Kaempferol-3-*O*-(6’’-*O*-Malonyl)-β-D-Glucopyranoside

Purification and NMR structural elucidation was undertaken for a major flavonol peak for which no commercial standards were available (**Figure 4H**), of particular interest due to its highly variable distribution pattern amongst individual accessions within the collection (**Figures 4A, B**). Based on UPLC-DAD-MS/MS analysis, this peak was suspected to be kaempferol-3-*O*-(6’’-*O*-malonyl)-β-D-glucopyranoside (**Figure 5**). Detailed review of previously published hops and beer-chemistry studies (Segawa et al., 2006; Aron et al., 2012; Farag et al., 2012; Tanaka et al., 2014; Dresel et al., 2016; Maietti et al., 2017) revealed trace amounts and tentative identification for this peak, along with its quercetin analogue, based solely on interpretation of UV-Vis and MS/MS fragmentation patterns; to our knowledge, no definitive isolation and/or structural characterization of this molecule from hops has ever been published. While LC-MS/MS is a powerful predictive tool for structural determination, the identity of the glycoside moiety (glucose vs. galactose), the site of glycosylation (3-OH vs. 7-OH, etc.) and location of the acylation site (6’’OH vs. 3’’OH, etc.) is not revealed by this type of analysis. While two previous reports of NMR structures for kaempferol-3-*O*-(6’’-*O*-malonyl)-β-D-glucopyranoside have been published, having been isolated from pears (*Pyrus communis*) and mulberry (*Morus alba*) (Wald et al., 1989; Lee and Choi, 2012), neither of these studies used comprehensive 2D NMR techniques to validate structural assignment of the acyl group nor the sugar moiety.

High-field NMR analysis (^1^H and ^13^C DEPTq135, gHSQC, gH2BC, gHMBC; **Supplementary File 1**) was used for validation of the kaempferol-3-*O*-(6’’-*O*-malonyl)-β-D-glucopyranoside structure (**Figure 5**), and summarized in **Table 4**. Our ^1^H and ^13^C assignments closely match previous reports (Wald et al., 1989; Lee and Choi, 2012). Examining the aromatic proton region, a typical AA’ BB’ ^1^H spin system was observed for B-ring protons at 7.98 ppm (2H, d, J = 8.9, H-2’,6’) and 6.88 ppm (2H, d, J = 8.9 Hz, H-3’,5’), along with two meta protons H-6 (6.21 ppm, d, J = 2.0 Hz) and H-8 (6.43 ppm, d, J = 2.0 Hz) on the A-ring, together confirming kaempferol as the aglycone. Using gHMBC, the anomeric proton (H-1’’) on glucose was observed to couple with C-3 of the kaempferol aglycone (133.1 ppm), confirming glycosylation at the 3-OH position. Similarly, a gHMBC between C-1’’’ of the malonyl moiety (166.6 ppm) and the 6-a’’/6-b’’ protons was observed, which clearly indicates esterification at the C-6’’ position as opposed to any other hydroxyl group on the sugar. The hexose sugar was identified as glucose using gH2BC to cycle around the pyranose ring identifying individual ^13^C nuclei, starting at C-1’’, with gHSQC assignment of corresponding proton signals in conjunction with comparisons of ^1^H coupling constants between adjacent protons, which all display *trans*- coupling with Js greater than 7.5 Hz. A β-glycosidic linkage was confirmed by *trans* J coupling (7.5 Hz) between (H-1’’ and H-2’’). Identification as D-glucose was confirmed using acidic hydrolysis of the glycosidic moiety, followed by *o*-tolyl isothiocyanate derivatization and analysis by UPLC-MS (Tanaka et al., 2007; Wang et al., 2012), in comparison to similarly derivatized D- and L- glucose and galactose reference compounds (**Supplementary Figure 1**).

**Table 4:** ^13^C (150.94 MHz), ^1^H (600.28 MHz) NMR spectroscopic data and important HMBC (^13^C with ^1^H) and H2BC (^1^H with ^1^H with ^13^C) correlations used to determine structure of kaempferol-3-*O*-(6’’-*O*-malonyl)-β-D-glucopyranoside, collected in DMSO-d_6_.

Position	δ^13^C (ppm)	δ^1^H (ppm)	*J* (Hz)	HMBC	H2BC
*kaempferol*					
*aglycone*					
2	156.4	–	–		
3	133.1	–	–		
4	177.3	–	–		
5	161.2	–	–		
6	98.7	6.21 d	2.0	103.9, 164.2, 156.7	
7	164.2	-	–		
8	93.7	6.43 d	2.0	103.9, 164.2, 161.2	
9	156.7	–	–		
10	103.9	–	–		
1'	120.7	–	–		
2' 6'	130.8	7.98 d	8.9	160.0, 156.4	
3' 5'	115.1	6.88 d	8.9	120.7, 160.0	
4'	160.0	–	–		
					
*β-D-glucose*					
1''	101.3	5.37 d	7.5	133.1	74.0
2''	74.0	3.19 dd	7.5, 9.2		76.1, 101.3
3''	76.1	3.23 dd	8.8, 9.2		69.5, 74.0
4''	69.5	3.14 dd	8.8, 9.5		73.9, 76.1
5''	73.9	3.32 ddd	9.5, 5.8, 1.9		69.5, 63.5
6a''	63.5	4.00 dd	11.8, 5.8	166.6	73.9
6b''		4.17 dd	11.8, 1.9		
					
*malonyl group*					
1'''	166.6	–	–		
2'''	41.3	3.08 s		166.6, 167.8	
3'''	167.8	–	–		

δppm, chemical shift in parts per million; s, singlet peak; d, doublet peak; dd, doublet of doublets peak; ddd, doublet of doublet of doublets peak.

## Conclusions

Controlled hybridization and introgression of quality traits between native North American *Humulus lupulus* ssp. *lupuloides* and European *Humulus lupulus* ssp. *lupulus* has been essential to the development of modern hops cultivars. Well-established breeding programs, either state-run or privately-operated, have generated dozens of registered cultivars in the past 50 years (USDA, 2007). New hops cultivars with unique flavor and aroma profiles drives innovation in the craft brewing industry, which has enjoyed continuous growth during the past decade, while conventional breweries have seen a contraction in sales volume due to changing consumer preferences. Developing regionally adapted hops cultivars and thereby tapping into the demand for locally produced foods and associated gastrotourism opportunities is essential to continued growth of the craft brewing sector.

Unfortunately, hops cultivars developed for warm and dry climates perform quite poorly in the eastern North America, requiring extensive applications of fungicides, insecticides and other agronomic cultural practices which contribute to environmental degradation and are cost prohibitive for producers. Additionally, yield potentials for most established hops cultivars show a marked decline when grown outside the PNW or Europe where they underwent initial breeding and selection trials (Darby et al., 2017). Sustainable and economically viable hops production in the New England and Maritimes areas of North America requires development of new, regionally-adapted and distinctive cultivars.

The *lupuloides* accessions surveyed and assembled into a germplasm repository during the course of this study are endemic to the Maritimes region of Canada, defined by its short, cool, and wet growing season (**Table 1**). When introduced to a research hopyard and subjected to commercial management practices, *lupuloides* accessions qualitatively show enhanced vigor, yield, and resistance characteristics, but additional years of quantitative measurement are required to establish commercial potential in a managed production setting (studies ongoing). The chemical composition of these *lupuloides* accessions supports utility in brewing, displaying alpha acid levels and ratios appropriate for use in popular American-style ales (**Table 2**). The essential oil compositions of these accessions have not been fully characterized, but show desirable and unprecedented aromas including melon, cucumber, herbal/spicy, and lemon (data not shown), which may be amendable to their application in other specialized brewing styles. The agronomic and chemical traits of these accessions possibly supports their outright development into named cultivars, or further use as a germplasm resource in hops breeding programs aimed at developing cultivars of the north east. Recently, two wild hops have been registered as novel varieties in North America, “Sasquatch”, selected from wild-growing accessions in British Columbia, and “Medusa”, selected from wild-growing *Humulus lupulus* ssp. *neomexicanus* plants in New Mexico, reflective of the value of our *lupuloides* accessions in future cultivar development.

The evolutionary history of hops in North America is under debate. While it is generally presumed hops migrated to North America from Asia at least half a million years ago following a cryptic coastal route, the divergence of this progenitor lineage into individual subspecies during the post-colonization era is highly speculative. *H. lupulus* ssp. *neomexicanus*, found in the south-western regions of the United States is well-adapted to arid conditions and often displays a diffuse, low-climbing growth habit, clinging to low bushes and grasses dominating the landscape, as opposed to the towering trees found in other regions. *H. lupulus* ssp. *pubescens*, predominantly found in the mid-west and upper Mississippi river valley region, is defined by profuse trichomes on abaxial leaf surfaces and shows limited ability to hybridize with other hops subspecies, perhaps due to incompatibilities in flowering time, with abundant trichome development hypothesized to act as a feeding deterrent against localized herbivores (Tembrock et al., 2016). *Humulus lupulus* ssp. *lupuloides*, found ranging from the northern Great Plains, through the Great Lakes, and into the Maritimes regions, displays less defined ecological specialization and morphological traits, sharing more characteristics with Asian (*H. lupulus* ssp. *cordifolius*) and European hops (*H. lupulus* ssp. *lupulus*) than the other subspecies. We hypothesize *H. lupulus* ssp. *lupuloides* most closely resembles the progenitor hops lineage that initially colonized North America, displaying a wider range of phenological, morphological, and chemical phenotype plasticity, allowing it to successful traverse the vast geographic distances spanning two continents. This versatility has allowed *H. lupulus* ssp. *lupuloides* to rapidly recolonize the northernmost extent of its range over the past 15,000 years following the most recent glacial retreat; perhaps doing so multiple times in the past half-million years of alternating interglacial periods.

**Figure 6:**
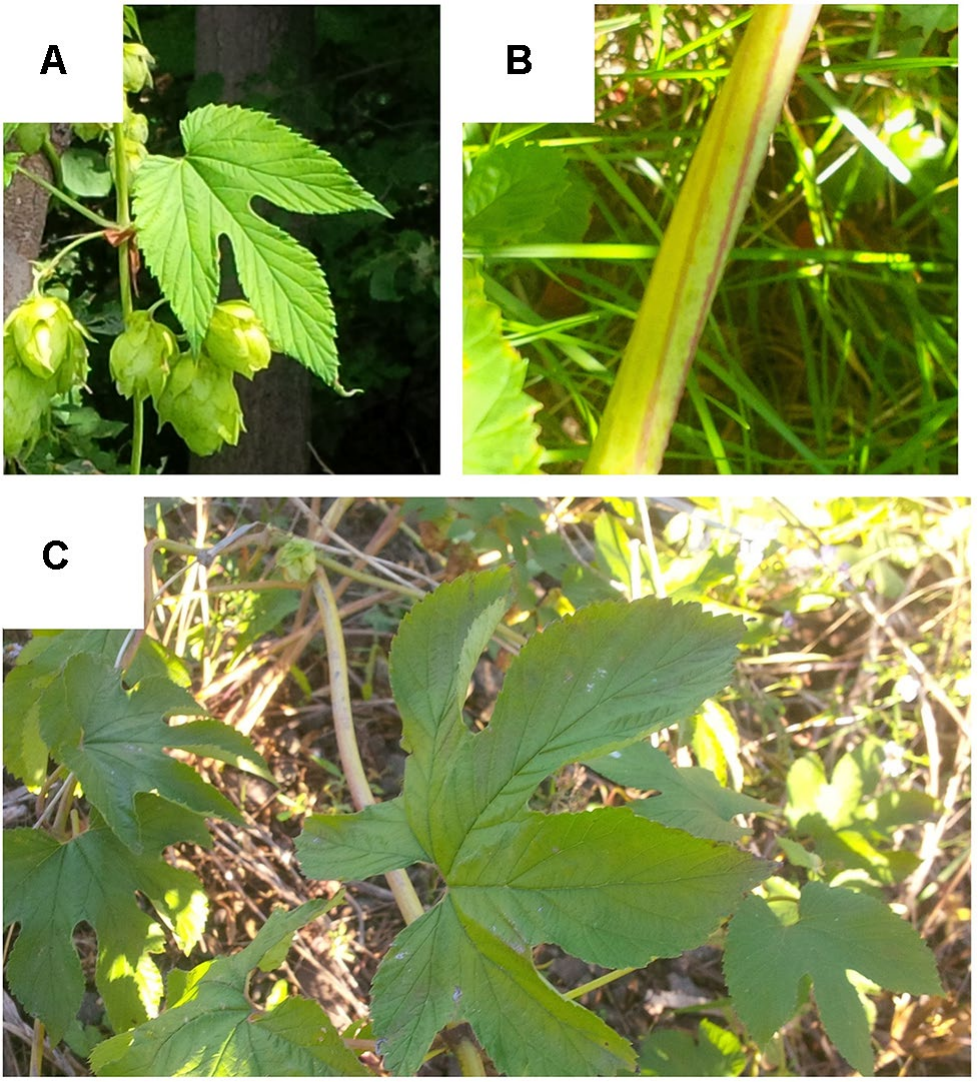
Some atypical phenotypic characteristics displayed by Maritimes *Humulus lupulus* ssp. *lupuloides* accessions. **(A)** elongated leaf blades; **(B)** “candy cane” striping of bines; **(C)** higher order leaf lobe number.

The *lupuloides* accessions we have assembled possess markedly higher levels of soft resins (bitter acids) and prenyl-chalcones than previously described for individuals studied in the Great Plains region. Additionally, these Maritimes region *lupuloides* accessions often display a number of atypical morphological traits, including elongated leaves, “candy-cane” striping of bines, higher leaf lobe numbers, and increased salt water tolerance, compared to what is typically reported for the subspecies (**Figure 6**). The impact of the Appalachian Mountains, a mostly north-south geographical divide parallel to the eastern coast of North America, on *H. lupulus* ssp. *lupuloides* evolution has not been fully explored. The Appalachian Mountains effectively isolate the Canadian Maritime provinces from the remainder of *H. lupulus* ssp. *lupuloides’* native range. Acting as a barrier to gene flow, this mountain range has a demonstrable effect on the frequency and distribution of molecular markers and genetic sequences in several species, ranging from trees and plants, fish, reptiles, and other aquatic vertebrates (Soltis et al., 2006; Jaramillo-Correa et al., 2009). A number of isolated and cryptic ice age refugia have been postulated to explain these genetic differences, with evidence of at least one of these areas located somewhere in the Northeast, perhaps on now submerged coastal islands exposed during the period of lower sea levels and vertical upwelling of the continental shelf due to downward thrust exerted by massive glaciers in the interior (Shaw et al., 2002; Soltis et al., 2006; Jaramillo-Correa et al., 2009). As climate conditions improved, the Maritimes region and New England states would presumably be repopulated by *lupuloides* from this coastal refugia, while the central Great Plains and Great Lakes region were recolonized by refugia on the western side of the Appalachians. Exploring how possible genetic drift and population bottlenecks have possibly affected divergence between eastern and western *lupuloides* populations is an emerging topic of interest. To answer these questions, further comparative studies focusing on molecular marker analysis between these populations, along with SNP analysis of critical developmental and biosynthetic genes will need to be conducted.

## Materials and Methods

### Hops Accessions

In 2015–2017, 61 wild-growing hops populations across the three Canadian Maritimes provinces were identified through a combination of herbarium records, botanical surveys (Catling, 1985), on the ground scouting of riparian zones and other public lands, along with input from interested members of the general public solicited through media outreach activities. Each hops population was characterized by GPS, topography, and environmental conditions noted, and site histories collected from informed land owners, where possible.

Early in the growing season (late June through early July), rhizome specimens from each site were collected using an ethanol sterilized spade, wrapped in damp paper towel for transport, and returned to laboratory facilities for propagation. Rhizomes were potted in Pro-Mix, incubated in greenhouse or growth chambers, and newly regenerated shoots subsequently excised, rooted in water (Stimroot; 0.1% indole-3-butyric acid), and repotted, or propagated through sterile tissue culture methods (Reed and Hummer, 2013), generating multiple somatic clones from each site for evaluation purposes. For each accession, a minimum of five clones were generated and maintained in greenhouse for planting at AAFC’s experimental hopyard (Harrington, PE, Canada), for continued evaluation of agronomic and chemical traits under commercial production conditions.

At the time of rhizome sampling, 5–10 mid-sized leaves (6–8 cm) in length were collected, as per Small (1978), pressed, and dried at 40°C, for later morphological and chemical characterization, while mature cones (50–800 g dried weight) were sampled later in the growing season (early September) at 20 sites for chemical analysis. At the time of manuscript preparation, a further 10 accessions have produced cones in the hopyard setting, for chemical evaluation purposes, bringing the total number of sites reported herein to 30 (**Figure 1B**, **Table 2**). Hops cones were dried to commercial levels (< 10% moisture), at 45°C using a combination of consumer-grade food dehydrators and an in-house forced air drier system, sealed in air tight plastic bags, and stored at −20°C for later analysis. For each individual harvest year and location, cones were analyzed in triplicate for chemical traits of interest, with the aggregate mean data for all years presented in **Table 2**.

### Laboratory Chemicals and Solvents

ACS-grade methanol for extraction, LC-MS Optima^TM^acetonitrile, and UHPLC-MS/MS Optima^TM^ grade solvents (water, acetonitrile, methanol) were from Fisher (Thermo Fisher Scientific, Nepean, ON, Canada). SupraPur^TM^ formic acid (98%–100%) and ACS grade isopropyl alcohol were from VWR (VWR International LLC, Mississauga, ON, Canada). Commercially available analytical standards were sourced from Extrasynthese (Genay, Cedex, FR) (quercetin-3-*O*-glucoside ≥ 99%; kaempferol-3-*O*-rutinoside ≥ 98%; quercetin-3-*O*-rhamnoside ≥ 98.5%) or Sigma-Aldrich (St. Louis, MO, USA) (quercetin-3-*O*-rutinoside ≥ 95%; kaempferol-3-*O*-glucoside ≥ 97%; xanthohumol ≥ 96%; quercetin-3-*O*-(6’’-*O*-malonyl)-glucoside ≥ 85%). International Calibration Extract 3 (ICE-3), an alpha & beta acid standardized mixture, was sourced from Labor Veritas (AG, Zurich, CZ). Deuterated solvent (DMSO-d_6_) was from CIL (Cambridge Isotope Laboratories Inc., Tewksbury, MA, USA). Pyridine, *o*-tolyl isothiocyanate, L-cysteine methyl ester, D-glucose, L-glucose, D-galactose, L-galactose were purchased from Sigma-Aldrich.

### Extraction Methods

#### Hops Cone Extractions

In brief, 2.5 g of dried hops cones (∼20 cones; 300 ml) were cryogenically ground to a fine powder using a mortar and pestle with liquid nitrogen, in triplicate. Aliquots of the powdered cones (0.5 g) were twice extracted in 25 ml of methanol:isopropanol (1:1), in a sonic bath with heating (35°C) for 30 minutes, centrifuged (2,000 xg), and successive extracts pooled for each sample. Samples were 0.45 µm GHP syringe filtered (VWR International LLC, Mississauga, ON, Canada), subsequent to UPLC-DAD-MS/MS analysis.

#### Hops Leaf Extractions

Dried leaves (∼1 g) were cryogenically ground in mortar and pestle with liquid nitrogen in triplicate, and portions (∼200 mg), twice extracted in 5 ml of methanol, in a sonic bath with heating (35°C) for 30 min, centrifuged (2,000xg), and successive extracts pooled for each sample. Samples were syringe filtered (0.45 µm GHP), subsequent to UPLC-DAD-MS/MS analysis.

### UPLC-DAD-MS/MS Characterization

UPLC-DAD-MS/MS (Acquity H-Class & TQD-MS, Waters Corporation, Milford, MA, USA) running MassLynx™ 4.1 was used to identify and quantitate individual alpha acids, beta acids, flavonols, and prenylchalcone compounds from dried hops cones and leaves. A Waters CSH™-C18 column (100 × 2.1 mm; 1.7 µm particle size) equipped with CSH™-C18 VanGuard™ pre-column (5 × 2.1 mm; 1.7 µm particle size), kept at 45°C, was used for all analysis. Samples were maintained at 10°C, injected *via* robotic autosampler (1 µl), and all mobile phase solvents filtered and degassed using an integrated inline degasser. For non-polar compounds (prenylchalcones, alpha & beta acids) the following linear gradient was employed: solvent A = water; solvent B = acetonitrile; solvent C = 10% formic acid in water; t0 A:B:C = 25:70:5; t4.5min A:B:C = 0:95:5; t6.25min A:B:C = 0:95:5; t7min A:B:C = 25:70:5; t8min A:B:C = 25:70:5, at a flow rate of 0.6 ml/min. For more polar compounds (flavonol glycosides), the same solvents were used according to the following linear gradient: t0 A:B:C = 93:2:5; t0.5min A:B:C = 93:2:5; t4.5min A:B:C = 65:30:5; t5.5min A:B:C = 0:95:5; t8.5min A:B:C = 0:95:5; t9min A:B:C = 93:2:5; t10min A:B:C = 93:2:5, at a flow rate of 0.45 ml/min. Both methods included a 5-min seal wash period, and have built in column cleaning and re-equilibration segments. Total diode array scans (230–700 nm) were collected at 20 Hz, with specific wavelengths chosen for quantitative analysis of individual components; 320 nm for alpha and beta acids; 370 nm for prenylchalcones; 350 nm for flavonols. For alpha and beta analysis, Hops International Calibration Extract 3 (ICE-3), with a defined composition (w/w) of 13.88% cohumulone, 30.76% n+adhumulone, 13.44% colupulone, 10.84% n+adlupulone was used. Individual prenylchalcones were quantified as xanthohumol equivalents, while individual flavonols were quantified as quercetin-3-*O*-rutinoside equivalents. Commercially available analytical standards were used to generate 7 point calibration curves prepared from three independent serial dilutions, plotting integrated area under the curve vs. concentration (mg/ml) yielding linear response curves (y = mx + b) with r^2^ values greater than 0.999, across 2.5 orders of magnitude (0.001–0.5 mg/ml).

For all samples, electrospray ionization (ESI) MS2 scans in both positive (+) and negative (−) modes were simultaneously monitored, from 200 to 1,500 amu with a scan time of 0.13 s. The following source conditions were used: capillary voltage 4 kV; cone voltage: 31 V in ESI+ or 15 V in ESI−; extractor 3 V; RF lens 1.9; source temperature 150°C; desolvation gas temperature 450°C; desolvation gas (nitrogen) flow rate 900 L/hr; cone gas (nitrogen) flow rate 5 L/hr. Using these conditions, the quasimolecular ions [M+H]^+^ or [M-H]^-^ were predominantly observed.

### Purification of Kaempferol-3-*O*-(6’’-*O*-Malonyl)-β-D-Glucopyranoside

A semi-preparative HPLC system running Empower 2.0 comprised of Waters 1525 Binary pump, Waters 2998 PDA, Waters 2707 autosampler and Waters Fraction Collector III, was used to isolate kaempferol-3-*O*-(6’’-*O*-malonyl)-β-D-glucopyranoside (aka malonyl astragalin), for which no commercial standard was available. In brief, a Waters XSelect^™^ CSH^™^ C18 prep column (150 × 10 mm, 5 µm particle size), equipped with a Waters XSelect^™^CSH^™^C18 prep guard-column (10 × 10 mm, 5 µm particle size), held at 60°C, was used under the following linear gradient at 2 ml/min: solvent A = water; solvent B = acetonitrile; t0 = 95% A; t1min = 95% A, t2min = 70% A; t10min = 67.6% A; t11min = 5% A; t13min = 5% A; t14min = 95% A; t16min = 95% A, with peak detection at 350 nm, triggered to collect by retention time window. A hops leaf methanolic extract (∼10 mg/ml total flavonols) enriched for the peak of interest was used as starting material, with 100 µl on column injected (full loop needle overfill = 300 µl) per chromatographic run, and with corresponding fractions from ∼65 successive runs combined. Acetonitrile was subsequently removed *via* rotary evaporation (Heidolph Instruments, Schwabach, Germany), and residual water removed *via* lyophilisation (LabConco Corporation, Kansas City, MO, USA), yielding 19.5 mg of light yellow powder in purity, which was characterized *via* 2D NMR methods.

### NMR Structural Elucidation of Kaempferol-3-*O*-(6’’-*O*-Malonyl)-β-D-Glucopyranoside

Approximately 5mg of kaempferol-3-*O*-(6’’-*O*-malonyl)-β-D-glucopyranoside was dissolved in 700-µl DMSO-d_6_ and put in a 5-mm NMR tube for NMR analysis. A Bruker Avance III 600MHz NMR spectrometer (Bruker Corporation, East Milton, ON) running TOPSPIN 3.5 software was used to acquire the NMR spectra using a 5-mm TCI Cryo-probe. Auto-tuning and shimming was performed on the sample at 600.21 MHz and 150.92 MHz for ^1^H and ^13^C, respectively. The π/2 pulse width for ^1^H was 8 and 12 µs for ^13^C.

For the ^1^H 1D, a 30° pulse sequence was used, a 20.02-ppm sweep width, an acquisition time of 2.73 s (64-K points), a 2.27-s relaxation delay, 4 dummy scans, and 32 scans for signal averaging. For 1D ^13^C information, a DEPTq135 was acquired with a 256.8-ppm sweep width, an acquisition time of 0.84 s (64-K points), a 2-s relaxation delay, 4 dummy scans, 8-k scans for signal averaging, and a ^1^J_CH_ of 145 Hz. The multiplicity edited gHSQC spectrum were acquired with an acquisition time of 0.056 s (1K points), 128 increments, a spectral width of 9 kHz in ^1^H and 36.2 kHz in ^13^C, 8 scans per increment, 16 dummy scans, and a ^1^ *J*_CH_ of 145 Hz was used for the INEPT transfers. The parameters for the gH2BC were the same as the gHSQC with the exception of 64 scans per increment were used. The gHMBC spectrum had an acquisition time of 0.17 s (3-K points), 200 increments, a spectral width of 9 kHz in ^1^H and 39.2 kHz in ^13^C, 256 scans per increment, 16 dummy scans, and the ^1^H–^13^C transfer was optimized for a ^2/3^ *J*_CH_ of 8 Hz while suppressing ^1^ *J*_CH_ of 145 Hz.

All spectra were processed and analyzed using the ACD/Spectrus Processor package (Advanced Chemistry Development, Inc., Toronto, ON, Canada). The determined structures gave excellent agreement with the simulated ^1^H and ^13^C spectra generated with the NMR prediction software add-on from ACD labs.

## Data Availability Statement

All datasets generated for this study are included in the article/**Supplementary Material**.

## Author Contributions

AM was responsible for plant propagation/maintenance, hopyard management, germplasm collection, along with project administration. SG performed phytochemical profiling, maintenance of plants, and germplasm collection. CK performed NMR structural elucidation and germplasm collection. MN performed phytochemical profiling and isolation of unknown compounds. JM performed phytochemical analysis, chemometric and morphological characterization of plants, germplasm collection, preparation of the manuscript, and project administration. All authors contributed to proofreading and approve the manuscript in its final form.

## Funding

This work was funded by AAFC A-base projects #1218 (J-001005) and #2963 (J-002214), developed and co-administered by JM and AM.

## Conflict of Interest

The authors confirm they have no conflicting financial interests in seeing this work published. Future development of commercial cultivars from these genetic resources is in progress, for release to Maritimes hops growers through Government of Canada licensing opportunities.

## Supplementary Material

Supplementary Figure 1 UPLC-DAD analysis of o-tolyl isothiocyanate derivatization products for D- and L-hexoside entantiomers, including hydrolyzed glycoside moiety liberated from kaempferol-3-O-(6’’-O-malonyl)-β-D-glucopyranoside.
