# Supplementary figures and images for: Phytochemical Characterization of Wild Hops (Humulus lupulus ssp. lupuloides) Germplasm Resources From the Maritimes Region of Canada
Source: Front Plant Sci. 2019 Dec 11;10:1438. doi: 10.3389/fpls.2019.01438 (PMC6917649; doi:10.3389/fpls.2019.01438)

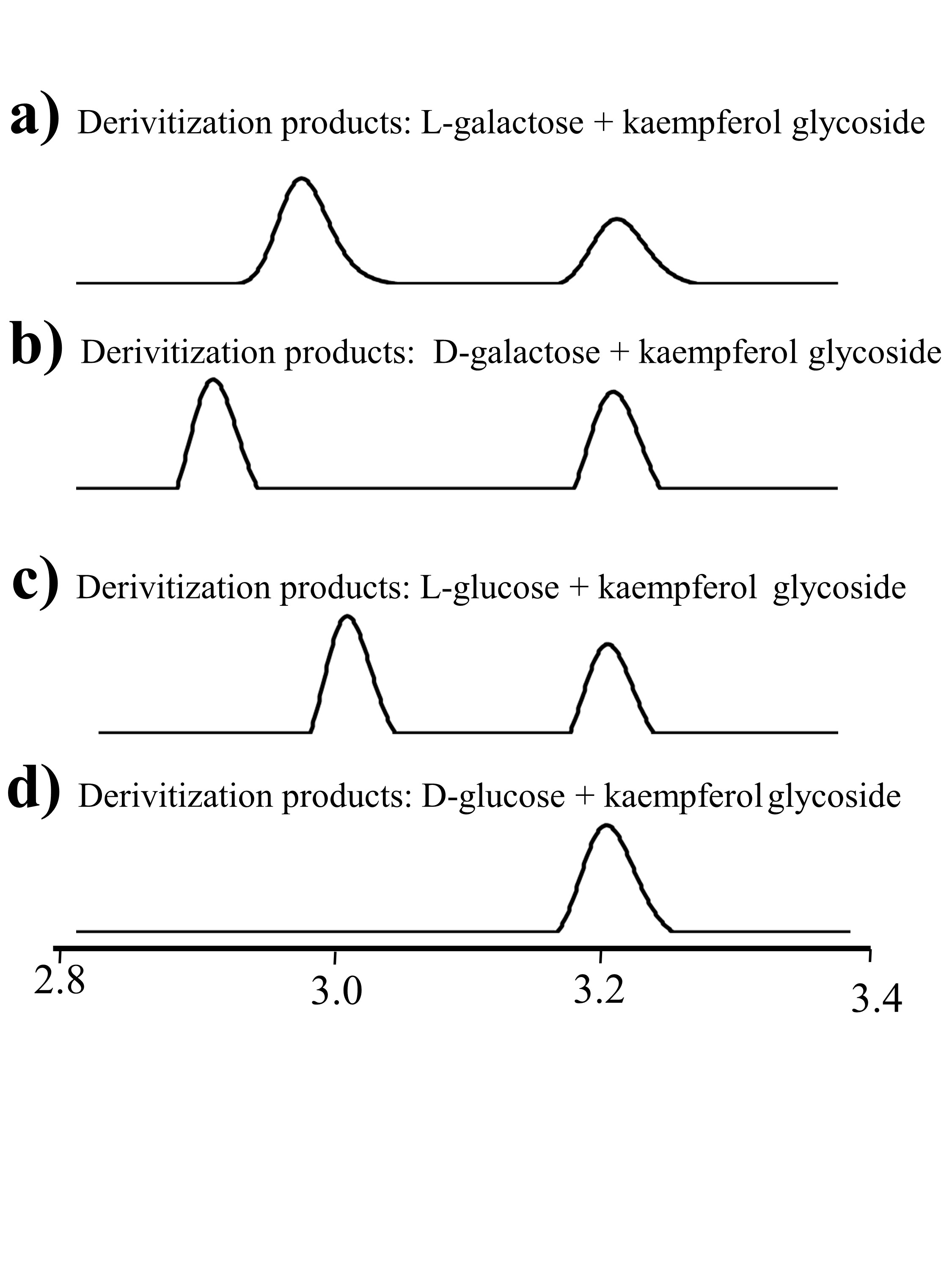

Supplement: Supplementary Figure 1 — UPLC-DAD analysis of o-tolyl isothiocyanate derivatization products for D- and L-hexoside entantiomers, including hydrolyzed glycoside moiety liberated from kaempferol-3-O-(6’’-O-malonyl)-β-D-glucopyranoside. [file Image_1.jpeg]
