# Supplementary material for: Phytochemical Characterization of Wild Hops (Humulus lupulus ssp. lupuloides) Germplasm Resources From the Maritimes Region of Canada
Source: Front Plant Sci. 2019 Dec 11;10:1438. doi: 10.3389/fpls.2019.01438 (PMC6917649; doi:10.3389/fpls.2019.01438)
Supplement: Supplementary file 2 [file Presentation_1.pptx]

## Slide 1
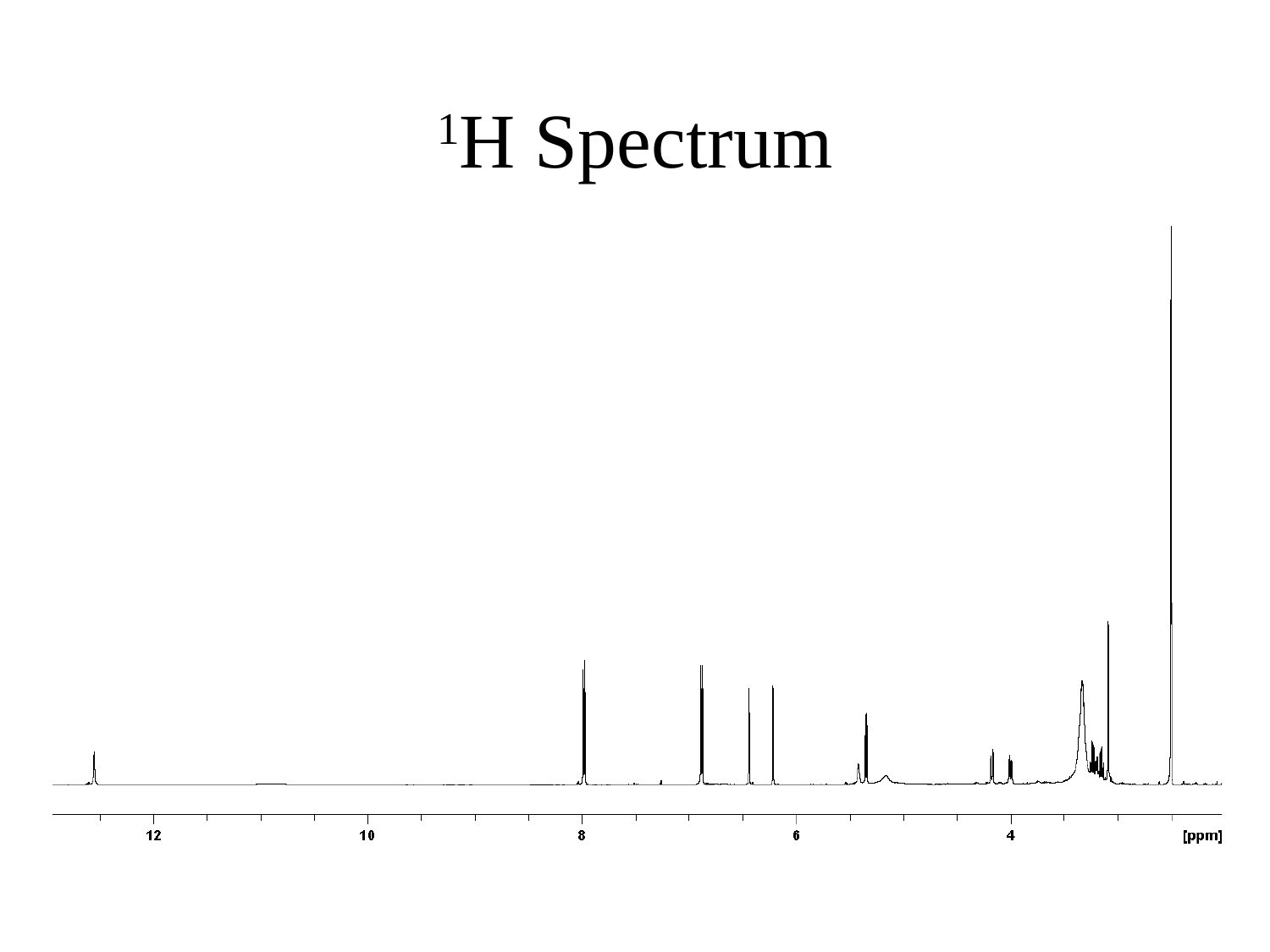

# 1H Spectrum

## Slide 2
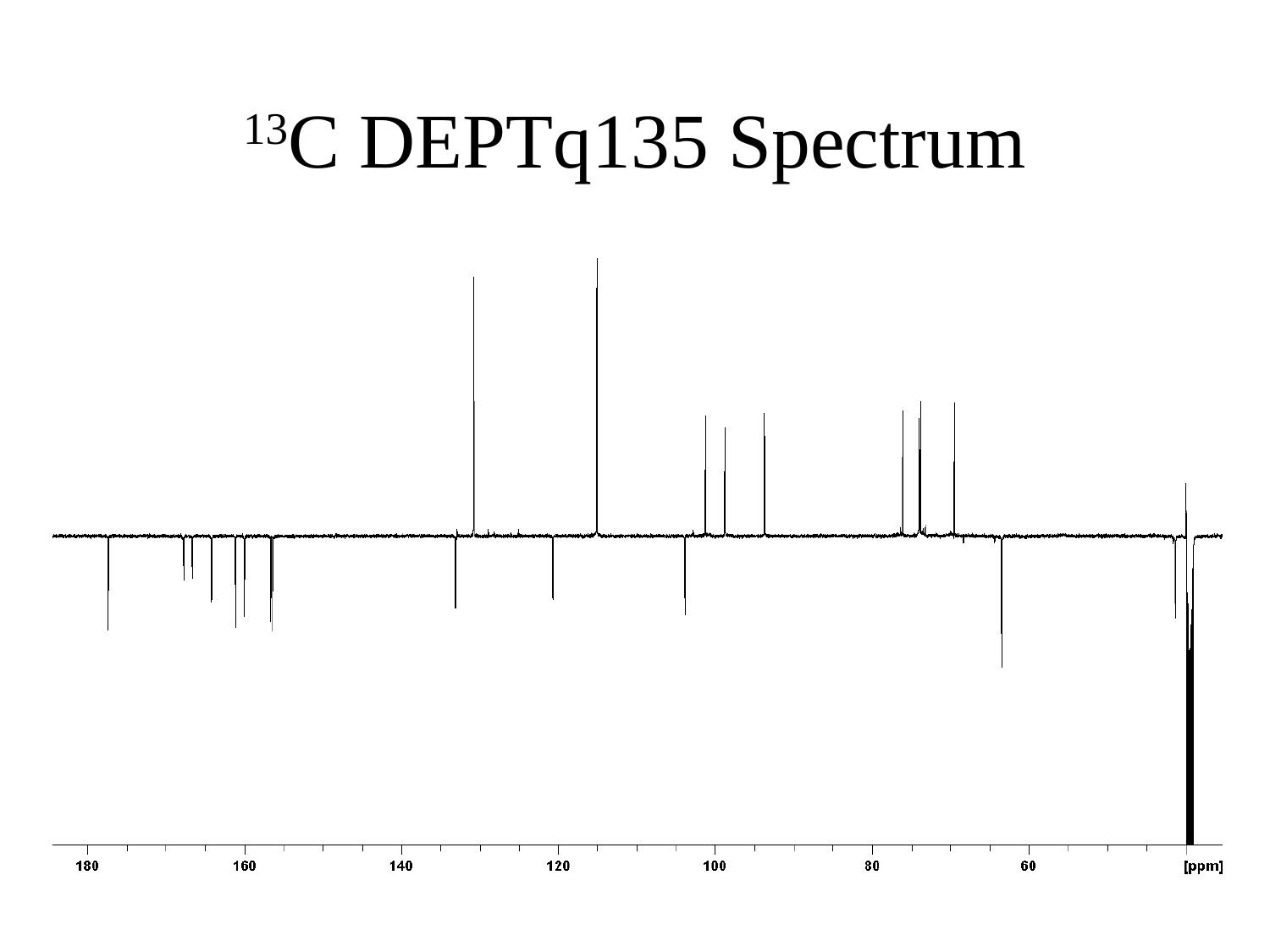

# 13C DEPTq135 Spectrum

## Slide 3
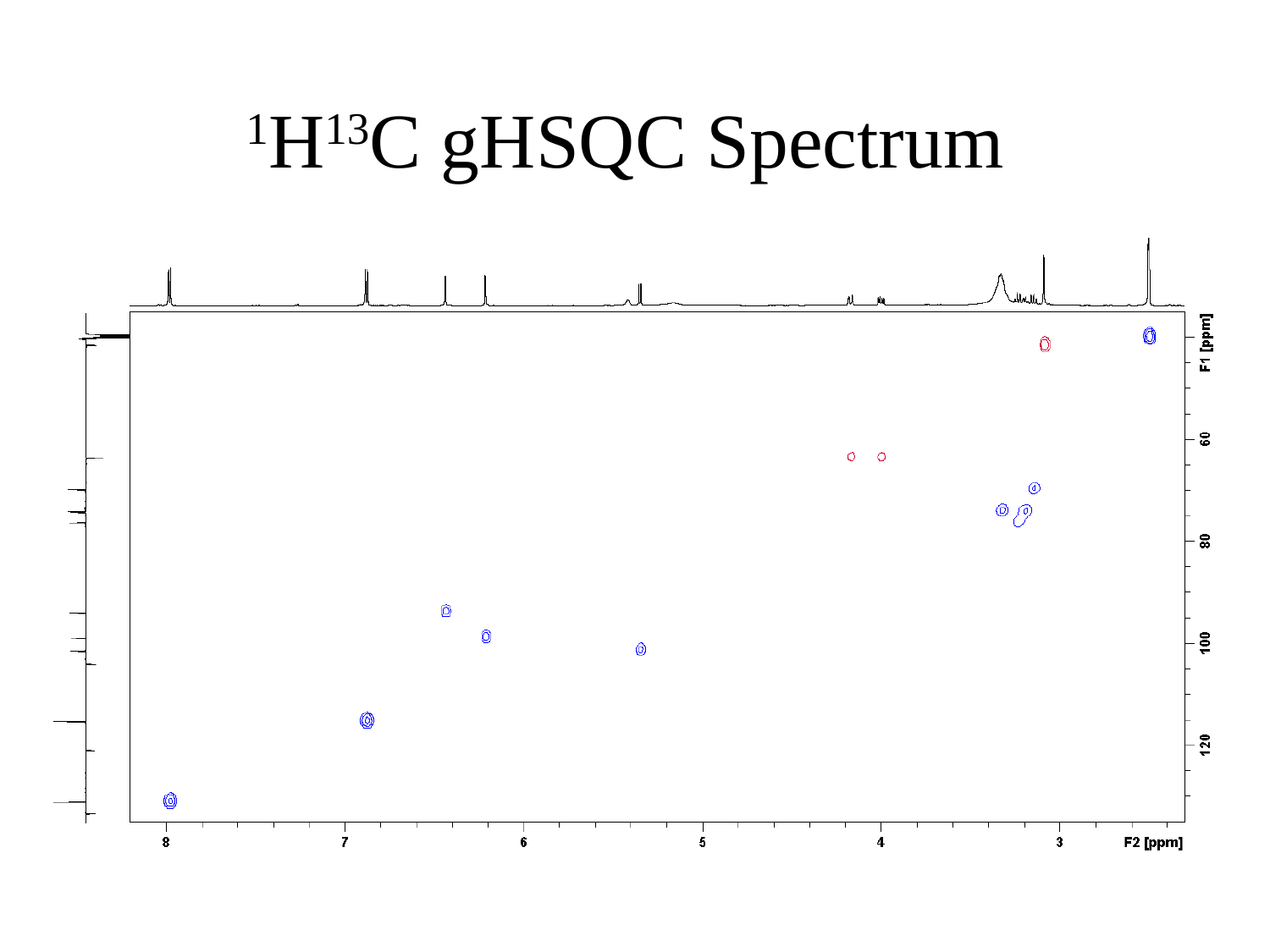

# 1H13C gHSQC Spectrum

## Slide 4
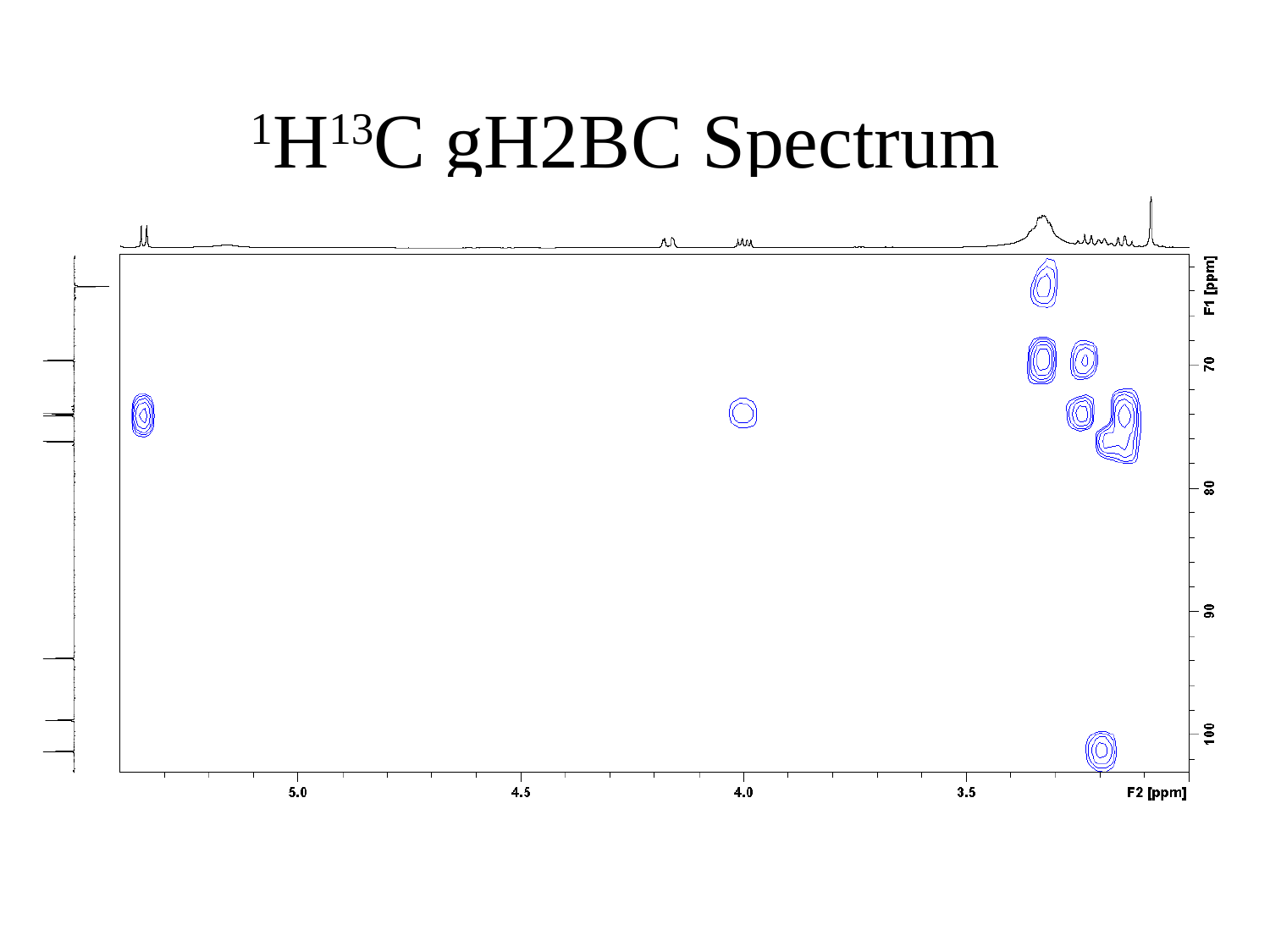

# 1H13C gH2BC Spectrum

## Slide 5
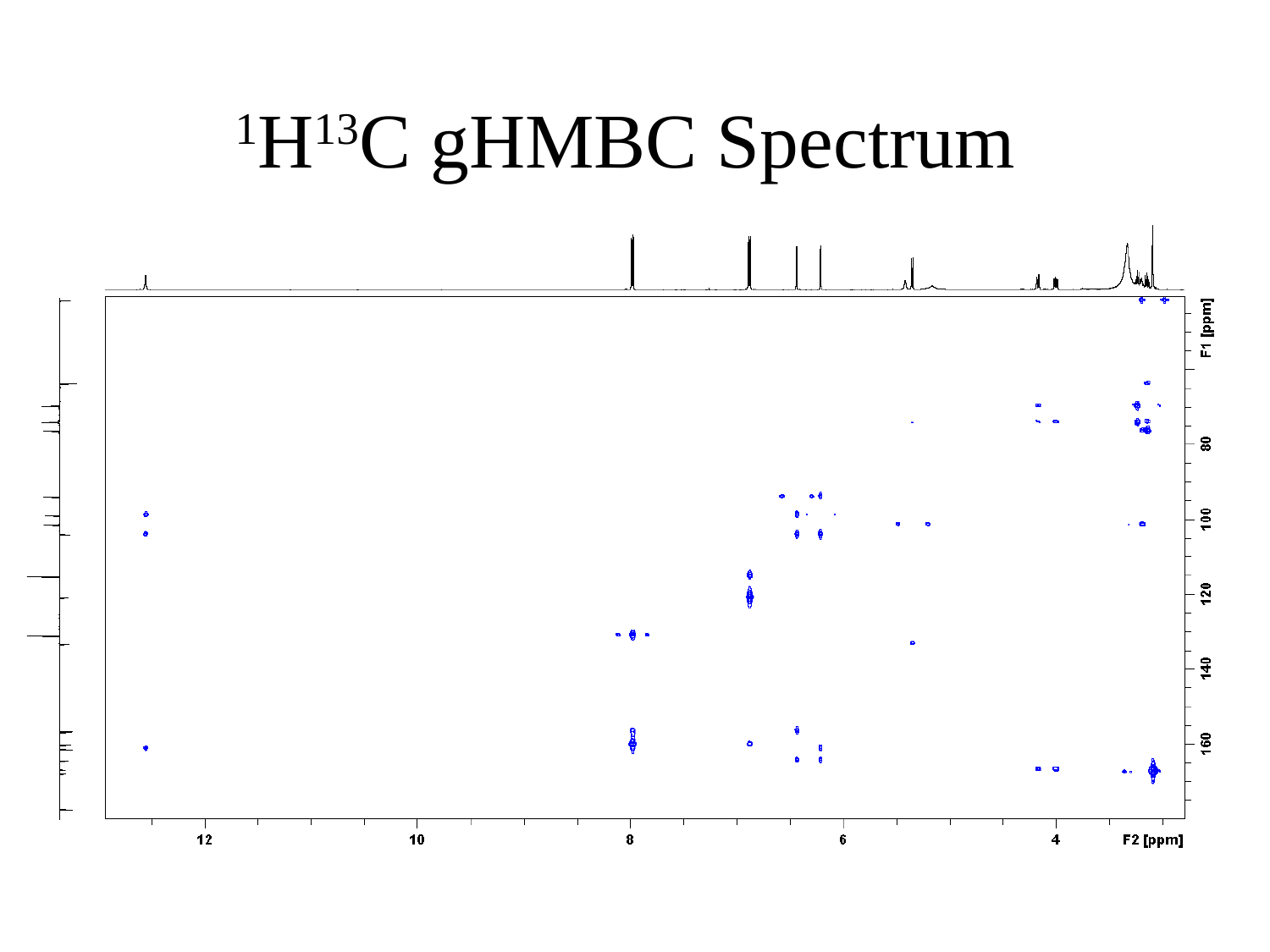

# 1H13C gHMBC Spectrum
